# Effect of Parietal Transcranial Magnetic Stimulation on Spatial Working Memory in Healthy Elderly Persons - Comparison of Near Infrared Spectroscopy for Young and Elderly

**DOI:** 10.1371/journal.pone.0102306

**Published:** 2014-07-14

**Authors:** Kaori Yamanaka, Hiroi Tomioka, Shingo Kawasaki, Yumiko Noda, Bun Yamagata, Akira Iwanami, Masaru Mimura

**Affiliations:** 1 Department of Neuropsychiatry, Keio University School of Medicine, Tokyo, Japan; 2 Department of Neuropsychiatry, Showa University School of Medicine, Tokyo, Japan; 3 Hitachi Medical Corporation, Application Development Office, Optical Topography Group, Tokyo, Japan; Maastricht University, Netherlands

## Abstract

In a previous study, we succeeded in improving the spatial working memory (WM) performance in healthy young persons by applying transcranial magnetic stimulation (TMS) to the parietal cortex and simultaneously measuring the oxygenated hemoglobin (oxy-Hb) level using near-infrared spectroscopy (NIRS). Since an improvement in WM was observed when TMS was applied to the right parietal cortex, the oxy-Hb distribution seemed to support a model of hemispheric asymmetry (HA). In the present study, we used the same study design to evaluate healthy elderly persons and investigated the effect of TMS on WM performance in the elderly, comparing the results with those previously obtained from young persons. The application of TMS did not affect WM performance (both reaction time and accuracy) of 38 elderly participants (mean age  = 72.5 years old). To investigate the reason for this result, we conducted a three-way ANOVA examining oxy-Hb in both young and elderly participants. For the right parietal TMS site in the elderly, TMS significantly decreased the oxy-Hb level during WM performance; this result was the opposite of that observed in young participants. An additional three-way ANOVA was conducted for each of the 52 channels, and a *P* value distribution map was created. The *P* value maps for the young participants showed a clearly localized TMS effect for both the WM and control task, whereas the *P* map for the elderly participants showed less significant channels and localization. Further analysis following the time course revealed that right-side parietal TMS had almost no effect on the frontal cortex in the elderly participants. This result can most likely be explained by age-related differences in HA arising from the over-recruitment of oxy-Hb, differentiation in the parietal cortex, and age-related alterations of the frontal-parietal networks.

## Introduction

Cerebral hemispheres are apparently structurally symmetric, but there are laterality in their function, a so called functional hemispheric asymmetry (HA). Starting from the pioneer work of Broca and Wernicke of aphasia, research study of HA has been developed drastically until we came to use high technology neuroimaging tool such as PET and fMRI. Among numerous studies in cognitive areas, Tulving et al. [1], [2] studied neuroanatomical correlates of encoding and retrieval processes of episodic memory as revealed by positron emission tomography (PET). They emerged the functional asymmetry in frontal cortex, and proposed the model of hemispheric encoding/retrieval asymmetry (HERA). Rossi et al. [3] used colored magazine pictures to investigate the model of HERA. They used TMS to transiently interfere with either left or right prefrontal brain activity during the task. The result showed that, during encoding, TMS over the left prefrontal cortex (PFC) disturbed the performance than TMS over the right PFC, whereas during retrieval, TMS over the right PFC disturbed the performance than TMS over the left PFC.

In our previous study, we demonstrated an improvement in the WM performance of healthy young subjects by applying TMS [4]. We applied TMS to either the left or right parietal cortex while the participants performed a WM task and a control task (a two-choice serial reaction time task). TMS was applied during the delay period of each task with a pulse of 5 Hz and an intensity of 100% of the resting motor threshold (rMT). The reaction time (RT) for the WM task was significantly shorter when TMS was applied to the right parietal cortex. The effect of TMS on the frontal cortex was also examined simultaneously by measuring the oxygenated hemoglobin (oxy-Hb) level using NIRS.

Referring to the previous numerous works, a model of HA seemed to be involved in the mechanism responsible for this improvement in healthy young participants [2], [5], [6], [7]. We suggested that the functional asymmetry of parietal cortex, remotely connected to the frontal cortex, indirectly influenced the working memory performance.

While the previously reported study was performed in healthy young participants, we have also used the same study design to evaluate healthy elderly participants and investigated the effect of TMS on the performance of a WM task and the oxy-Hb. Since aging is associated with declining cognitive function [8], [9], [10], change in the brain structure [11], [12], [13], [14], and neurotransmission [15], we hypothesized that an age-related difference in both the performance and oxy-Hb movements related to HA may exist in the elderly, while we anticipated that the WM would also improve similar to the effect observed in the young participants.

## Materials and Methods

### Ethics Statement

This study was approved by the Ethics Committee of Showa University School of Medicine. All the participants gave written informed consent in accordance with the Declaration of Helsinki after a complete explanation of the study.

In this study, we used the same study design that were used in our previous paper [4], mainly for task design, TMS application and NIRS measurements.

### Participants

#### Young participants

We used data obtained for participants who participated in a previous study [1] for comparison purposes. Fifty-two healthy persons (30 men and 22 women) with a mean age of 23.4±2.7 years were recruited as paid volunteers. All the young participants were screened for depression using the Zung Self-rating **Depression** Scale **(SDS)** [16]; those who scored 40 or above were excluded from the present study. All the participants were right-handed and had normal or corrected-to-normal vision. Candidates were excluded if they had a history of neurological or psychiatric disorders, including substance abuse/dependence. Participants were randomly divided into two groups, depending on the parietal sites for TMS. Two sites, P3 in the left parietal cortex and P4 in the right parietal cortex, were chosen for stimulation in the previous study, based on the international 10–20 system [17]. TMS was applied to the P3 and P4 regions in 27 and 25 participants, respectively.

#### Elderly participants

Thirty-eight healthy elderly persons (18 men and 20 women) with a mean age of 72.4±4.7 years (range, 64 to 83 years) were recruited as paid volunteers in the present study. All the participants were right-handed and had normal or corrected-to-normal vision. Candidates were excluded if they had a history of neurological or psychiatric disorders, including substance abuse/dependence. Instead of screening using the SDS, all the participants were screened for depression using the Japanese version of the Geriatric Depression Scale **(GDS)** [18]. We used GDS instead of SDS because we determined the reliability and validity was superior to SDS for the elderly participants. SDS has a several problem for elderly participants. Mean score for elderly participants tends to be higher than that of younger participants, which induces a false positive [19]. Another problem is that SDS has a multiple choices that may be confusing to elderly participants. GDS only has “yes/no” choices which may be more acceptable for elderly participants. Those who scored 5 or above were excluded from the present study. All the participants were screened for dementia using the Mini-Mental State Examination (MMSE) [20], and those who scored 24 or below were excluded from the present study. As in the previous study, 38 elderly participants were randomly divided into two groups, depending on the parietal sites for TMS. TMS was applied to the P3 and P4 regions in 18 and 20 participants, respectively (Table 1.).

**Table 1:** Participation information for the young and elderly participants.

	Participant Information	
	Young	Elderly
N	52	38
N (female)	22	21
Mean age (SD)	23.5 (2.7)	72.5 (4.7)
Minimum age	20	64
Maximum age	30	83
SD = standard deviation		

### Tasks

**Figure 1:**
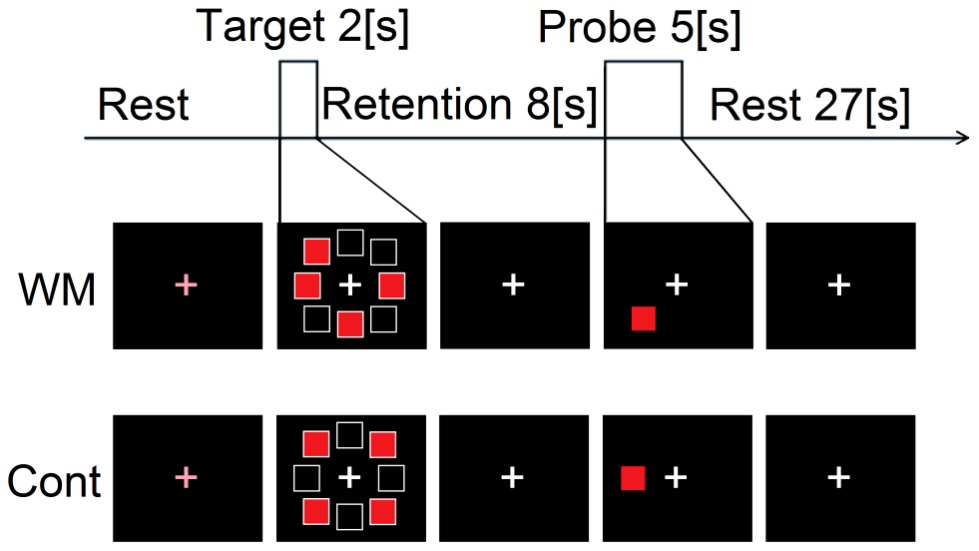
Example of the tasks used in the study. Upper row: working memory task. Lower row: control task. The upper row requires a “no” response, and the lower row requires a “left” response. The trial phases and their durations are shown at the top. The arrow indicates the duration of TMS.

Participants were trained to perform two delayed match-to-sample tasks (a spatial WM task and a control (Cont) task), which were presented on a personal computer (Inspiron XPS M1710; Dell, USA). Each trial lasted for 42 s and followed a predetermined sequence of phases (Figure 1). Participants sat on an upright chair in front of a monitor, which was located about 50 cm from the subject's eyes. The participants were requested to perform the spatial WM task or the Cont task and either a real TMS or a sham TMS was administered during each task. The participants repeated ten trials under each of the four sets of conditions (i.e., WM-Real, WM-Sham, Cont-Real, or Cont-Sham). The order was counterbalanced across the participants, and a 10-min break was provided between each condition. Participants received sufficient training before the actual NIRS measurement so that they were able to perform the task adequately (i.e., >80% correct responses). They were instructed to respond as quickly and as accurately as possible.

#### Spatial WM task

The trial commenced with a black background and a central fixation image (white cross) that turned pink as a warning signal. After a warning interval (2 s), four red squares were presented at peripheral locations (four out of a possible eight locations) as a target cue (2 s), which was then followed by a delay period of 8 s. Subsequently, a red square was presented as a probe cue at one of the eight peripheral locations for 5 s. The participants were requested to report whether the location of the probe cue was identical to the location of any of the target cues based on spatial WM. The ‘yes’-‘no’ response was given by pressing a button with the right index finger or the left index finger, respectively. Each trial was followed by an inter-trial interval (ITI) of 25 s, during which time a white cross was presented in the center of the display. Throughout the trial, the participants were instructed to maintain visual fixation on the central cross. The ‘yes’ and ‘no’ trials were pseudo-randomized to be equal in number (i.e., five ‘yes’ trials and five ‘no’ trials).

#### Control (Cont) task

An identical target cue consisting of red squares at four of the eight peripheral locations was presented after a warning interval, followed by a delay period of 8 s. Then, a red square was presented as a test cue at either the right or left side of the screen and the participants were requested to press the corresponding button. The ‘right’ and ‘left’ trials were pseudo-randomized to be equal in number. This control task reproduces almost all the features of WM task such as stimuli and attentional resources, but much easier than the WM task.

### TMS

**Figure 2:**
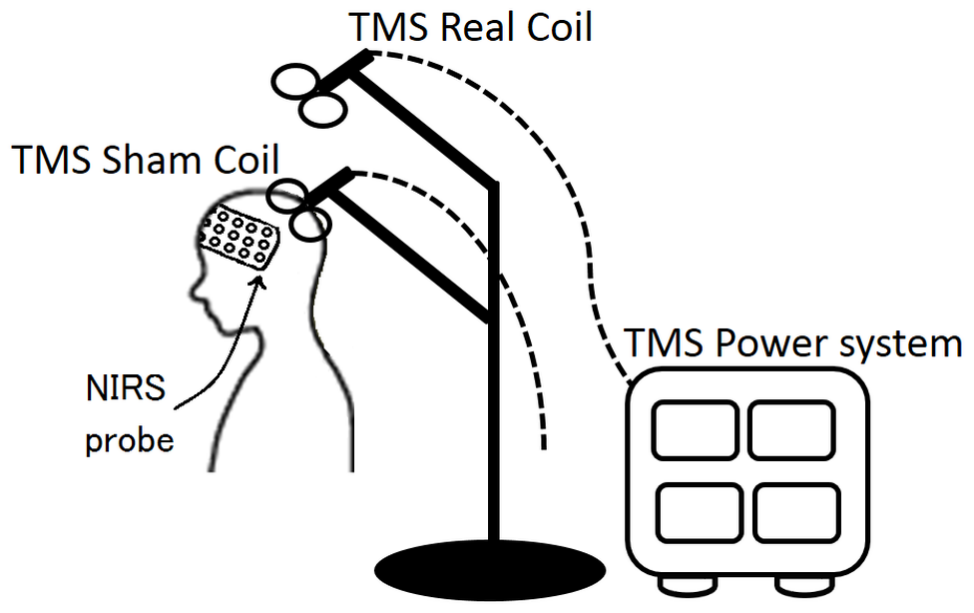
TMS Sham condition. The illustration shows how the Sham TMS is applied. A pseudo TMS is applied to the subject's parietal cortex, while the other TMS, connected to the power source, is fixed 30 cm above the subject's head. For the Real TMS condition, the TMS that is applied to the subject's head is connected to the power source.

Both real and sham TMS were performed. Real TMS was applied using an air-cooled figure-eight coil (70 mm in diameter) powered by a Magstim Rapid System (Magstim Co. Whitland, UK). For the “sham TMS”, the coil was disconnected from the power supply but was placed on the participant's head in the same manner as during the real TMS. To create the sound and the vibration of TMS without actually delivering a magnetic stimulus, the active coil was placed on the steel coil holder about 30 cm above the scalp (Figure 2). The participants were told that the coil would have different outputs **so** that they would not guess whether the coil was real or a sham.

The TMS stimulus intensity was set at 100% rMT for the right hemisphere, which was defined as the lowest intensity needed to evoke motor potentials of at least 50 µV recorded from the first dorsal interosseus muscle after at least 5/10 stimuli. Thirty pulses of TMS were applied manually at 5 Hz over the delay period for 6 s with an 100% rMT, according to the published safety guidelines [21]. A total of 300 pulses (30×10) were given during each session, which lasted for 7 min and 40 s.

At the end of each four sessions, we asked the participants to guess whether each condition was real or a sham (i.e. Four questions for each subjects). Of all sessions throughout the participants, only11.9% were correctly distinguished whether the coil was real or sham. Therefore, the sham condition, i.e., the vibration generated by the real coil placed on the steel coil holder and transmitted to the sham coil, appeared to have successfully produced similar acoustic cues and skin sensations.

### Statistical analysis of task data

The accuracy (mean number of correct responses) and the RT (interval between presentation of the probe cue on the screen and the subject's response) of each trial were determined for each participant. The responses were scored as errors in the case of a wrong response or no response during the 5-s inter-stimulus interval. These data were then grouped across the participants for analysis, with RT and accuracy being analyzed separately.

An independent sample T-test was performed to compare the mean RT separately for the elderly and the young in each condition (WM-Real, WM-Sham, Cont-Real, Cont-Sham).

For each group (young, elderly), a three-way repeated-measures analysis of variance (ANOVA) was performed to assess the accuracy and the RT according to the stimulation site (P3 or P4) as a between-subject factor and the task (WM or Cont) and TMS (Real or Sham) as within-subject factors. The **s**ignificance was set at *P*<0.05 for all the comparisons.

### NIRS

#### Measurement of Hb

NIRS images were obtained using a 52-channel NIRS system (ETG-4000; Hitachi Medical Corporation, Tokyo, Japan). The changes in the oxy-Hb, deoxy-Hb, and total Hb concentrations were measured using two different wavelengths of near-infrared light (695 and 830 nm) to detect oxy-Hb and deoxy-Hb. The distance between the pair of emission and detector probes was 3.0 cm, and the machine was thought to measure the conditions at a depth of 2–3 cm from the scalp, i.e., the surface of the cerebral cortex [22], [23]. The probes of the NIRS machine were fixed with thermoplastic 3×11 shells, with the lowest probes positioned along the Fp1–Fp2 line according to the international 10–20 system used for electroencephalography. The 52 measuring areas were labeled ch1-ch52 from the right-posterior to the left-anterior. The correspondence between the NIRS channels and the measurement points on the cerebral cortex was confirmed using a multisubject study of anatomical craniocerebral correlation [24] and was displayed based on the results of a virtual registration method [25].

Raw optical data for the two near-infrared frequencies were recorded simultaneously from each of the 52 channels every 100 ms and were sent to a data collection computer via an analog-digital converter. The timing of each task event (e.g., the onset of the sample cue) was also transmitted to the data collection computer from the task control computer.

#### Statistical analysis of Hb

**Figure 3:**
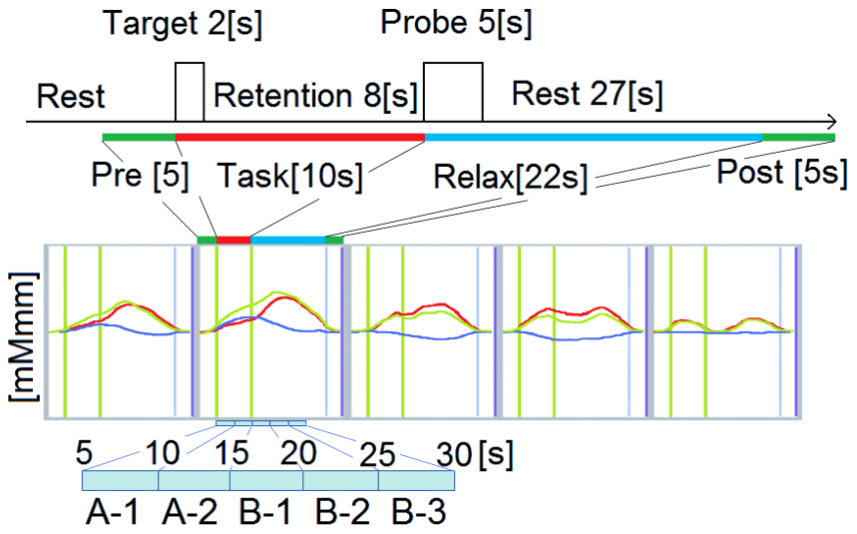
Diagram of the task and the corresponding NIRS time distribution. Data were analyzed using the “integral mode” to obtain the average of the pre-task baseline and the post-task baseline (top row, green line). Linear fitting was applied to the data between these two baselines. The moving average method was used to exclude short-term motion artifacts. The oxy-Hb data from each channel were averaged across the initial 25 s of the activation period (task + first 15 s of relax), which was divided into five time segments of 5 s each (A-1: earlier phase of maintenance, A-2: latter phase of maintenance, B-1: first phase of reaction, B-2: second phase of reaction, and B-3: third phase of reaction).

Before we performed our previous study for the young participants, we conducted a preliminary test for young volunteers using NIRS, to estimate the approximate hemodynamic response time needed, after the task until the wave converges to the baseline(relax time). Average time needed for the relax time was approximately within 22∼23 seconds. To analyze the raw optical data, we first defined a 32-s period from the onset of the target cue as the activation period [task (10 s) + relax (22 s)] (Figure 3). Second, we defined 5-s periods before and after the activation period as the pre-activation and post-activation baseline periods, respectively. Then, linear fitting was applied to the data between these two baselines. The moving average method was used to remove short-term motion artifacts from the data (moving average window: 5 s). After averaging each condition over 10 trials, oxy-Hb was calculated using the modified Beer–Lambert law. We focused on oxy-Hb because it was assumed to reflect cognitive activation more directly than deoxy-Hb, as shown by its stronger correlation with the blood oxygenation level-dependent signal on fMRI [26]. For data analysis using parametric statistical tests, the oxy-Hb data from each channel were averaged across the first 25 s of the activation period [task (10 s) + relax (first 15 s)], which was divided into five time segments. We did not use the last 7 s of the relax time because of the potential artifact. The length of each segment was 5 s (A-1: earlier phase of retention, A-2: latter phase of retention, B-1: first phase of reaction, B-2: second phase of reaction, B-3: third phase of reaction).

An independent sample T-test was performed to compare the mean oxy-Hb of the elderly and the young participants for each condition (WM-Real, WM-Sham, Cont-Real, Cont-Sham). To clarify the effect of TMS on each task and the task x TMS interaction effect, a three-way repeated-measures ANOVA for oxy-Hb was performed with the stimulation site (P3 or P4) as a between-subject factor and the task (WM or Cont) and TMS (Real or Sham) as within-subject factors. Furthermore, the data were split into each channel (Ch: 52 levels), and a three-way repeated-measures ANOVA was separately performed for the oxy-Hb data from each Ch. The statistical analysis was performed using SPSS 16.0 J for Windows software (Tokyo, Japan).

## Results

None of the participants reported adverse events, and no seizures occurred during the experiment. RTs that were longer than three standard deviations from the group mean were regarded as outliers and were excluded from the analysis.

### Task performance

To determine whether there was a trade-off between RT and accuracy, possible correlations were evaluated. No significant correlations were observed across all the conditions for both young and elderly participants (all *P* values >0.05).

**Figure 4:**
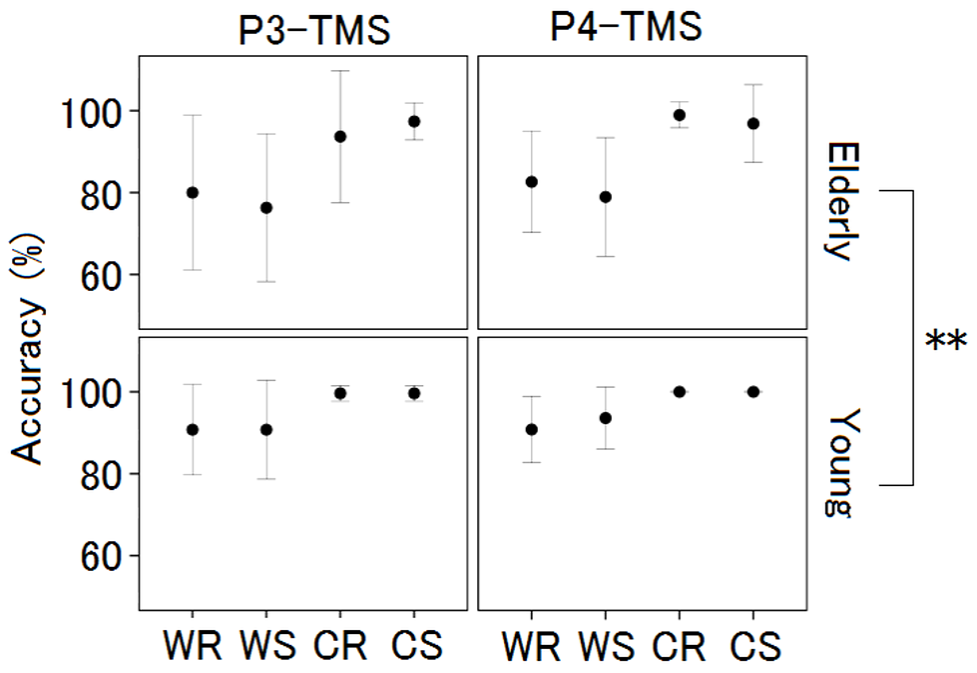
Mean accuracy for young and elderly participants. No interaction effect was observed when a three-way (site × task × TMS) ANOVA for accuracy was performed. The accuracy was poorer for the elderly participants for all the conditions (WM Real, WM Sham, Cont Real, and Cont Sham). WR: WM Real, WS: WM Sham, CR: Cont Real, CS: Cont Sham. The error bars show the standard error of the mean.

The accuracies for each group (young and elderly) were as follows: young (WM-Real: 90.77%, WM-Sham: 92.12%, Cont-Real: 99.81%, and Cont-Sham: 99.81%) and elderly (WM-Real: 81.32%, WM-Sham: 77.63%, Cont-Real: 96.32%, and Cont-Sham: 97.11%). The accuracies were was poorer in the elderly group than in the young group for all the conditions (WM Real, WM Sham, Cont Real, and Cont Sham). A three-way ANOVA with 2 (Site; P3 vs. P4) × 2 (Task; WM vs. Cont) × 2 (TMS; Real vs. Sham) repeated measures for each group did not show any interactions influencing the performance accuracy (Figure 4).

**Figure 5:**
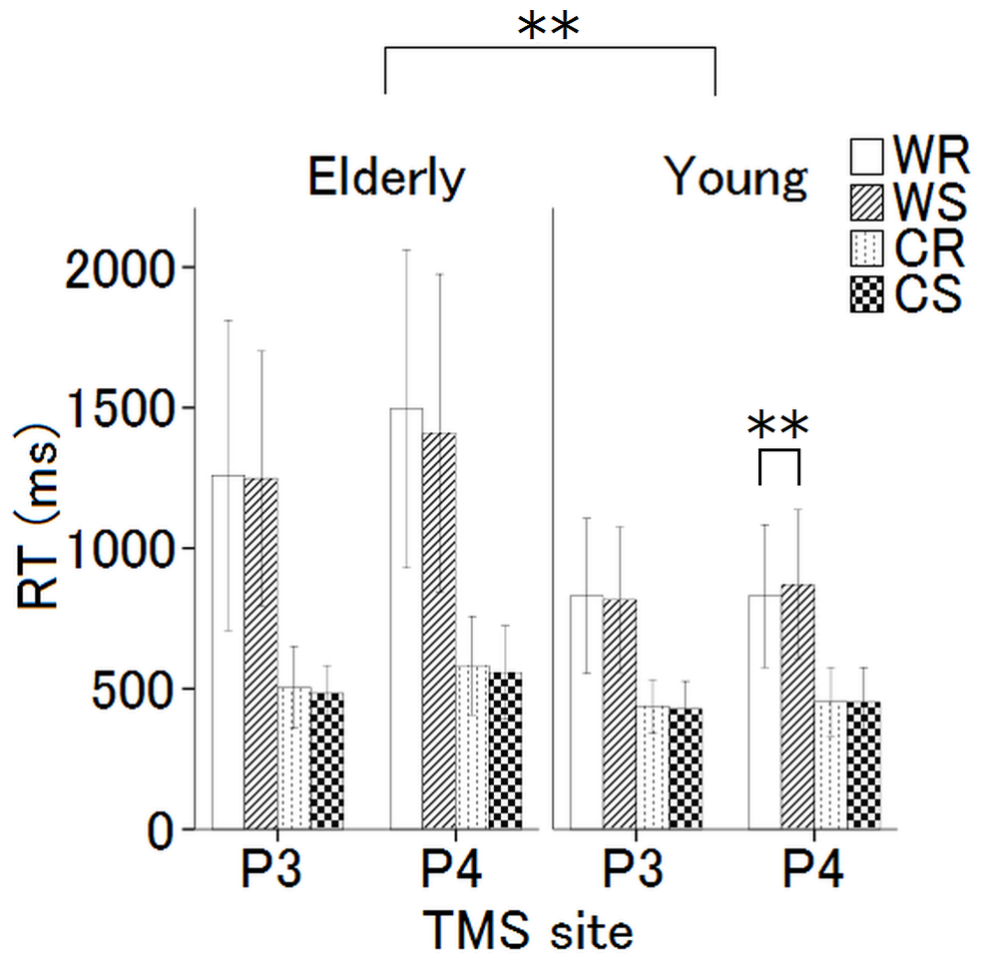
Mean RT for the young and elderly participants. The mean RT of the elderly participants was significantly longer than that for the young participants for all the conditions (WM Real, WM Sham, Cont Real, and Cont Sham). The application of TMS to P4 in the young participants was the only condition under which the RT of WM was improved. The error bars show the standard error of the mean.

The mean RT was compared between the young and elderly participants. The mean RT for the elderly participants was significantly longer than the RT for the young participants for all the conditions (WM-Real, WM-Sham, Cont-Real, Cont-Sham) (*P*<0.01). A three-way ANOVA for RT with 2 (site, P3 vs. P4) × 2 (task, WM vs. Cont) × 2 (TMS, real vs. sham) repeated measures for young and elderly participants was conducted (Figure 5). A post hoc analysis showed that TMS over P4 site during WM task was the only condition that RT was significantly shortened (p = 0.06; P4WMReal:819 ms, P4WMSham:879 ms).

For the RT of the elderly, the main effect of task (F_1, 204_ = 832; *P*<0.01), and the interaction effect of site x task (F_1, 204_ = 4.18; *P* = 0.042) were significant, while the main effect of TMS (F_1, 204_ = 3.58; *P* = 0.06) and the interaction effects of task x TMS (F_1, 204_ = 1.25; *P* = 0.27), TMS x site (F_1, 204_ = 0.84; *P* = 0.36), and site × task × TMS (F_1, 204_ = 0.81; *P* = 0.37) were not significant. A post hoc analysis showed that main effect of site was significant (p<0.01; P3:887 ms, P4:1011 ms) and none of interaction effect was significant. From these results indicated that, mean RT on P3 site was significantly shorter than that on P4 site, and the mean RT for the WM task was longer than that for the Cont task, irrespective of TMS.

### Changes in oxy-Hb

**Figure 6:**
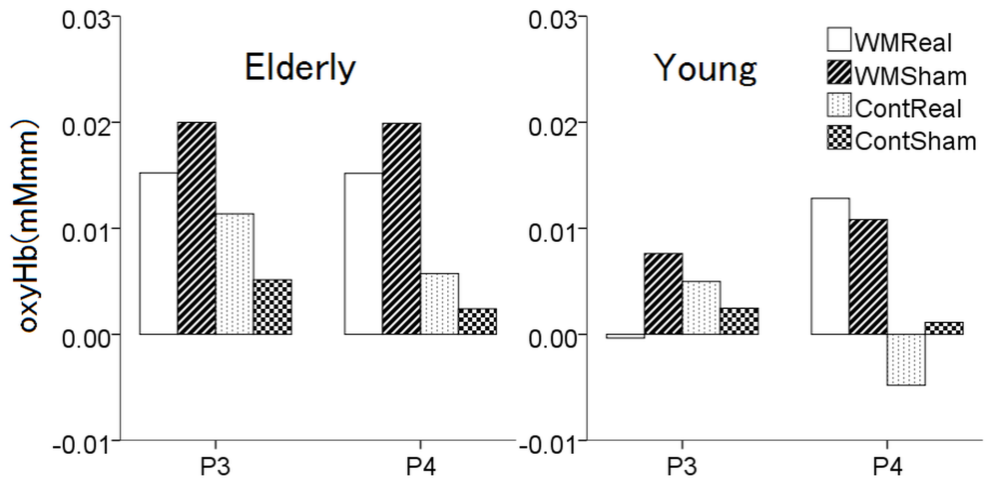
Oxy-Hb results for the young and elderly participants. A three-way (Site × Task × TMS) ANOVA was separately conducted for the young and elderly participants. The Site × Task × TMS interaction effect was significant for the young participants (*P*<0.05). Of note, the application of TMS to P4 in the young participants was the only condition under which the oxy-Hb increased during the the WM Real (Real > Sham) and decreased during the Cont Real decreased (Real < Sham). The application of TMS to P4 in the young participants was the only condition under which the RT improved.

Oxy-Hb levels that were longer than 3 standard deviations from the group mean were excluded from the analysis as outliers. The mean amplitude of the oxy-Hb change was compared between the young and elderly participants. The amplitude of the oxy-Hb change was larger for the elderly participants than for the young participants under all the conditions (WM-Real, WM-Sham, Cont-Real, and Cont-Sham) (*P*<0.01). Focusing on the effects of TMS, a three-way repeated measures ANOVA for the oxy-Hb data with 2 (site, P3 vs. P4) × 2 (task, WM vs. Cont) × 2 (TMS, real vs. sham) repeated measures was conducted (Figure 6).

For the oxy-Hb data in the young participants, as shown in our previous paper [1], a three-way repeated-measures ANOVA showed a significant main effect of Task, TMS, and an interaction effect of Site × Task × TMS. For the next step, we divided the data according to the Site factor and performed a two-way repeated-measures ANOVA with 2 (task, WM vs. Cont) × 2 (TMS, real vs. sham) repeated measures separately for P3 and P4. For TMS at P3, the result indicated that real TMS caused a decrease in oxy-Hb during the WM task, whereas TMS increased oxy-Hb during the Cont task. For TMS at P4, the main effect of Task, TMS, and an interaction effect of Task × TMS were significant. A post-hoc analysis revealed that with TMS at P3, oxy-Hb significantly decreased during the WM task, whereas oxy-Hb significantly increased during the control task. Conversely, with TMS at P4, oxy-Hb significantly increased during the WM task and significantly decreased during the control task.

For the oxy-Hb data obtained in the elderly participants, a three-way repeated-measures ANOVA showed a significant main effect of Task (F_1, 9868_ = 651.7; *P*<0.01) and interaction effects of Site × Task (F_1, 9868_ = 21.1; *P*<0.01) and Task × TMS (F_1, 9868_ = 156.9; *P*<0.01). The main effect of TMS (F_1, 9868_ = 0.001; *P* = 0.979) and the interaction effects of Site × TMS (F_1, 9868_ = 3.21; *P* = 0.073), and Site × Task × TMS (F_1, 9868_ = 3.69; *P* = 0.055) were not significant. A post-hoc analysis revealed that with TMS, oxy-Hb significantly “decreased” during the WM task, whereas oxy-Hb significantly “increased” during the Cont task for both TMS sites (*P*<0.01). When the effect of TMS was compared between the elderly and the young, only the young participants with TMS at P4 site exhibited a unique result, in which oxy-Hb was significantly increased while performing the WM task and oxy-Hb was significantly decreased while performing the Cont task.

**Figure 7:**
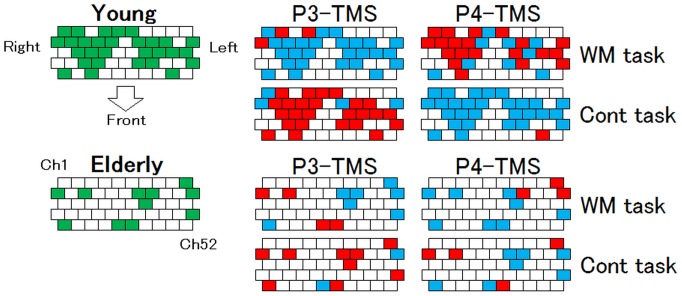
Distribution of significant *P* values mapped on the NIRS channels. The results of a three-way (Site × Task × TMS) ANOVA for oxy-Hb conducted for each of the 52 channels are show for both the young and the elderly participants. The channels for which the Site × Task × TMS interaction effect was significant are shown. The green channels indicate a location where the *P* value <0.05. The red channels indicate channels for which the oxy-Hb was increased, and the blue channels indicate channels for which the oxy-Hb was decreased.

To see the effect of TMS on each NIRS channel, an additional three-way (Site × Task × TMS) ANOVA was conducted for the 52-channels data obtained for the young and the elderly participants separately. Site × Task × TMS interaction effects on oxy-Hb were detected, as shown in Figure 7. The figures show the *P* value map for the 52 channels. The green channels indicate channels with a *P* value <0.05 for both the young and the elderly participants. The red channels indicate channels with a *P* value <0.05 in which oxy-Hb “increased”, whereas the blue channels indicate a “decrease”. On the P3 map for the young participants, the majority of significant channels for the Cont task increased and the majority of significant channels for the WM task decreased, while the significant channels on the P4 map for the young participants exhibited an opposite pattern to that for P3. On the other hand, both the P3 and P4 maps for the elderly participants showed less-significant channels and less-clear relations than those observed for the young participants.

**Figure 8:**
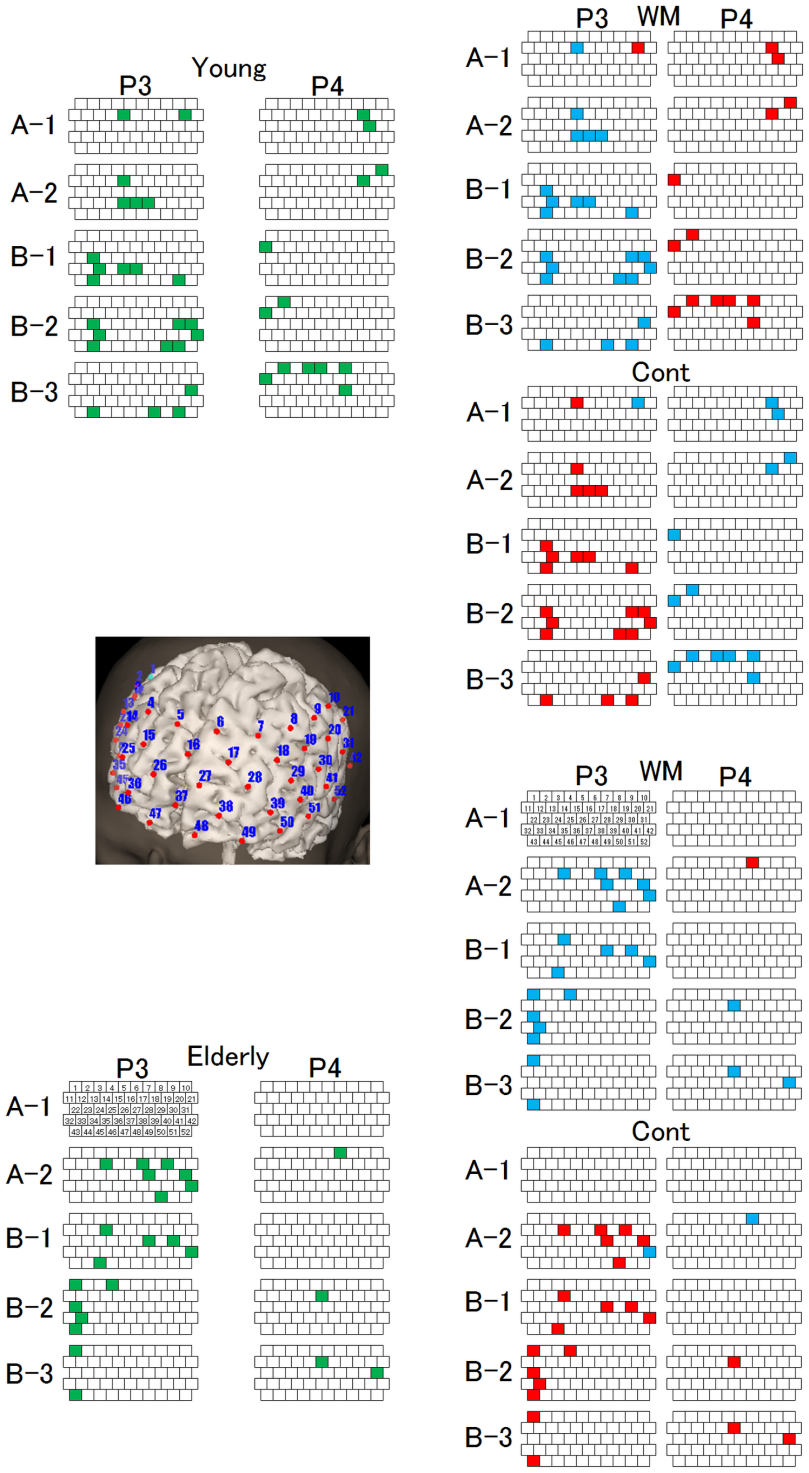
Distribution of significant *P* values mapped on the NIRS channels with time segments. The results of a two-way (Task × TMS) ANOVA for oxy-Hb conducted for each TMS site, 52 channels, and time segment are shown for both the young and the elderly participants. The channels for which the Task × TMS interaction effect was significant are shown. The green channels indicate a location where the *P* value <0.05. The red channels indicate channels for which the oxy-Hb was increased, and the blue channels indicate channels for which the oxy-Hb was decreased. Notice that almost no significant channels for the elderly P4 TMS condition are present for all the segments.

To further analyze the time course changes for each site, we conducted a two-way (task × TMS) ANOVA separately for the TMS sites (P3, P4), time segments (A-1, A-2, B-1, B-2, B-3), and the 52-channels data obtained for the young and the elderly participants (Figure 8). On the map for the young participants, the oxy-Hb channels decreased during the WM task for almost all the segments with P3 TMS, whereas all the segments showed an increase during the WM task with P4 TMS. The relative relation between the tasks was also maintained. On the map for the elderly participants, the oxy-Hb channels decreased during the WM task for almost all the segments with P3 TMS, whereas almost no significant channels were observed throughout the task with P4 TMS.

## Discussion

In the present study, the application of 100% rMT-TMS at 5 Hz to the parietal cortex of elderly participants during the delay period of a WM task did not affect the WM performance, either in terms of RT or accuracy, while the application of TMS to the right parietal cortex (P4) did improve the performance of young participants in terms of the RT for the WM task. Here, we discuss the difference between the findings for young and elderly participants and the reason for this difference.

### Over-recruitment of Oxy-Hb

The mean RT of the elderly participants was significantly longer for all the conditions (i.e., WM-Real, WM-Sham, Cont-Real, and Cont-Sham) than the mean RT for the young participants (Figure 5). This finding suggests that the elderly participants required a longer time to perform the task regardless of the TMS application. We observed a delay in the elderly, compared with the young participants, even for the control task, which may only reflect the time required to perform the various attention tasks. However, for the RT of the WM task, an even greater delay was observed, suggesting the existence of a delay in the information storage portion of WM as well as a delay in the simple motor response.

The mean oxy-Hb level of the elderly participants was also significantly higher than that of the young participants under all the conditions (Figure 6). This result suggests that the elderly participants consumed a larger quantity of oxy-Hb during the task regardless of the TMS application. Functional neuroimaging studies of the effects of age on the neural correlates of working memory have reported that, relative to young participants, older individuals demonstrate greater and more widespread cortical activity. Cabeza (2002) [27], [28] proposed that over-recruitment reflects the involvement of additional neural resources that compensate for the age-related decline, in order to achieve the same performance out comes as younger adults. Reuter-Lorenz and Cappell, 2008 [29]also proposed a similar hypothesis known as “*Compensation Related Utilization of Neural Circuits Hypothesis (CRUNCH)*” that older adults need to recruit more neuronal resources even at lower loads than younger adults, thus leads to performance decrements at a higher loads [30]. In this study, the task performances neither improved nor worsened in terms of accuracy and RT, but the oxy-Hb was significantly higher than in the young participants. On the map of the *P* values for oxy-Hb shown in Figure 7, most of the significant channels that were affected by TMS overlap with the channels observed in the young participants, but some different cortical areas have also been recruited, such as Ch 47 and 48.

**Figure 9:**
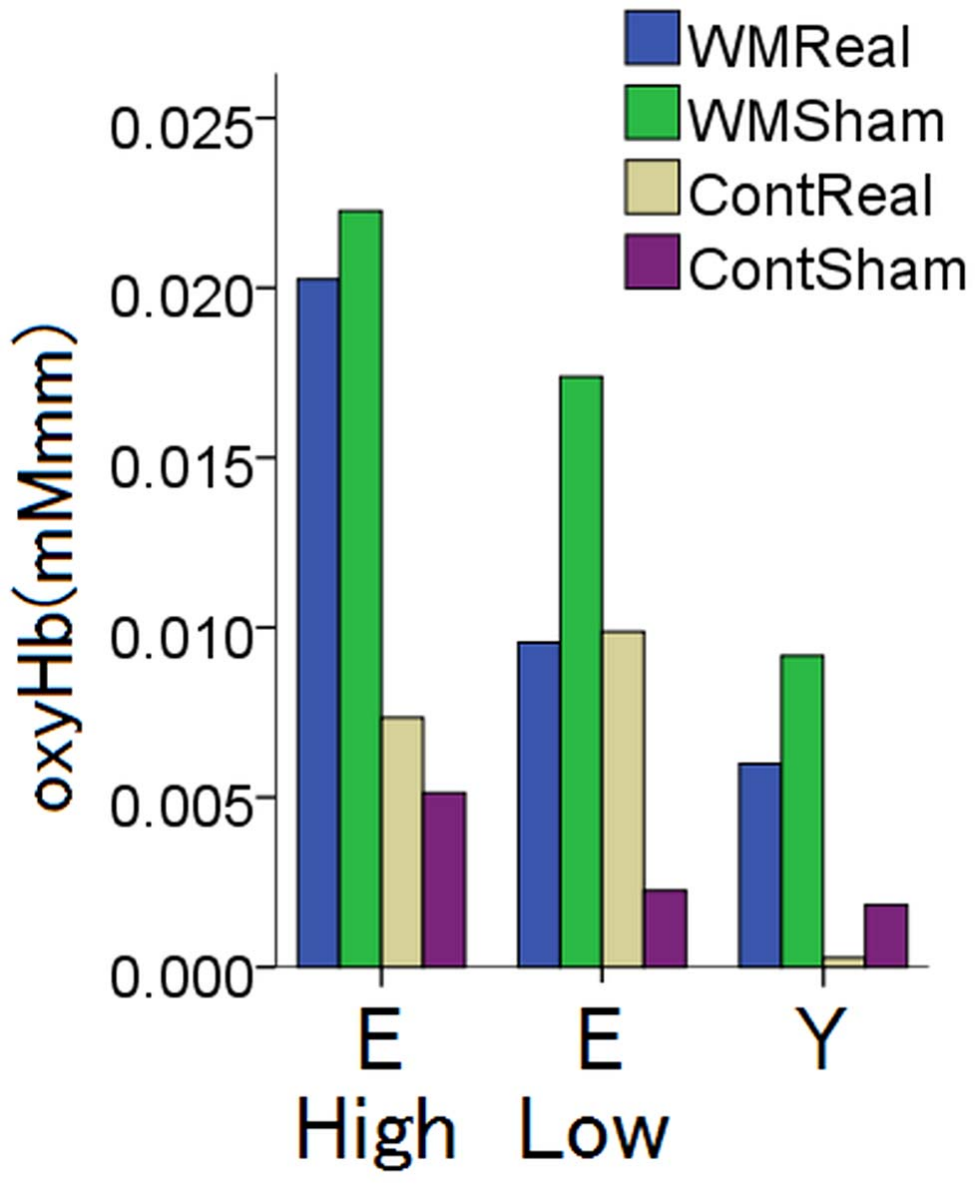
Oxy-Hb of high and low performers. Oxy-Hb was compared between high performers with an accuracy of 80% or more for all conditions (WM Real, WM Sham, Cont Real, and Cont Sham) and low performers with an accuracy of less than 80% for at least in one condition. E: Elderly, Y: Young, High: high performer, Low: Low performer.

On the other hand, Nyberg reported cross-sectional and longitudinal analysis of structural aged brain changes using functional MRI (fMRI). They performed a second MRI session after six years using the same participants and compared the brain structures. They claim that the cross-sectional analyses suggested age-related frontal over-recruitment, whereas the longitudinal analyses revealed frontal underrecruitment with advancing age. They concluded that a relatively high-performance elderly sample biased the cross-sectional results in the direction of frontal overrecruitment. Additionally, we split our data into high performers with an accuracy of 80% or more for all conditions (WMReal, WMSham, ContReal, and ContSham) and low performers with an accuracy of less than 80% for at least in one condition and compared the magnitude of the oxy-Hb levels in the elderly with those in the young participants (Figure 9). A one way ANOVA revealed the oxy-Hb rankings as high performer >low performer>young performer (*P*<0.01). These results suggest that additional oxy-Hb was recruited to achieve the same performance outcomes as younger adults, but further longitudinal analysis might be needed to follow underrecruitment with advancing age.

### Differentiation of Parietal Cortex and an Alteration of Parietal-frontal Network

As seen in Figure 6, for the elderly participants, oxy-Hb significantly decreased for the WM task, whereas oxy-Hb significantly increased for the Cont task with either site of TMS. On the other hand, for the young participants, oxy-Hb significantly increased while performing the WM task and oxy-Hb significantly decreased while performing the Cont task with P4 TMS. We concluded that this HA in the young was the key to the improvement in the RT observed in the previous study [4]. Thus, we assumed in the present study that an age-related alteration of HA in the elderly brain may account for the results obtained here.

Further analyses elucidated the distribution of significant oxy-Hb changes in the frontal cortex. The data for the young participants showed a clear opposite relation of oxy-Hb within the task level and the TMS level, whereas the data for the elderly participants rarely showed such a relation (Figure 7). As expected, an age-related HA seemed to be involved in the results.

In fact, when we performed a two-way ANOVA for the elderly participants by splitting the data into two TMS sites, five time segments, and 52 channels, we were able to observe that there were almost no significant cannels with P4 TMS, indicating the rarity of a TMS effect on the frontal area (Figure 8). When we observed the effect of P3 TMS, there were as many significant channels in the elderly as observe in the young participants, and the relative relation of oxy-Hb between the WM and the Cont task was maintained, although the significant areas changed.

In investigating spatial WM in older and younger adults using fMRI, Piefke et al. (2010) [31] observed a similar age-related differentiation of the laterality of precuneus activation, while performing spatial WM task [32], [33], They observed right hemispheric activation in younger participants while they observed bilateral activation in older participants. Bäckman et al. (2011) [34] performed an fMRI study in young and elderly while the participants performed a spatial WM task under two levels of load. On a separate occasion, the same participants underwent PET measurements to determine the dopamine D1 receptor binding potential in the caudate nucleus and dorsolateral prefrontal cortex (DLPFC). The fMRI study revealed a significant amplitude difference of brain activity depending on the load, in the frontal and parietal regions for the young, but not in the elderly. The PET measurements revealed significant age-related reductions in the D1-binding potential in the caudate and DLPFC. They concluded that age-related changes in dopaminergic neurotransmission may contribute to the MRI result in the elderly. MacEvoy et al. (2001) [35] recorded the EEGs from healthy young, middle-aged, and elderly adults, while the participants performed a spatial WM task under two levels of load. The RT increased with age and accuracy decreased in the high memory load task relative to that in the low load task. Each age group revealed a different amplitude and latency of P300 and P200 component of the event-related potential in parietal and frontal regions, which suggest that normal aging may be associated with changes in the fronto-parietal networks involved with spatial WM processes.

With these results and numerous previous works handling the age-related changes, we assumed that a differentiation of the parietal cortex and an alteration of the parietal-frontal network existed in the elderly participants.

Our study was limited by its small sample size (elderly: n = 38, young: n = 52); however, the participants were rigorously screened. Since NIRS can only perform measurements on the surface of the brain, the differences and similarities in oxygenation as measured using NIRS need to be verified using other modalities, such as fMRI and PET.

## Conclusions

In the present study, WM performance was not enhanced in healthy elderly participants by the application of TMS to the parietal cortex. This result can most likely be explained by age-related deficits in HA arising from the age-related over recruitment of oxy-Hb, differentiation in the parietal cortex, and alterations of the frontal-parietal networks. However, this is the first study, to our knowledge, to investigate age-related changes in the WM mechanism using both TMS and NIRS. We successfully elucidated the brain functions in healthy elderly participants, which clearly differ from those in healthy young participants.
